# 2-(4-Chloro­phen­yl)-5-(3,4-dimethoxy­pheneth­yl)-6,7-dihydro­pyrazolo[1,5-*a*]pyrazin-4(5*H*)-one

**DOI:** 10.1107/S1600536809020212

**Published:** 2009-06-06

**Authors:** Jin-Hua Zhang, Hua Zuo, Yong-Sheng Xie, Bao-Xiang Zhao

**Affiliations:** a School of Chemistry and Chemical Engineering, Shandong University, Jinan 250100, People’s Republic of China; b College of Pharmaceutical Sciences, Southwest University, Chongqing 400716, People’s Republic of China

## Abstract

In the title compound, C_22_H_22_ClN_3_O_3_, the dihedral angles between the planes of the benzene rings and the pyrazole ring are 16.05 (10) and 84.84 (10)°. The conformation of the six-membered heterocyclic ring is close to a screw-boat. The crystal packing is stabilized by weak inter­molecular C—H⋯O inter­actions and is also consolidated by C—H⋯π inter­actions.

## Related literature

For the bioactivity of pyrazole derivatives, see: Farag *et al.* (2008 ▶); Pan *et al.* (2008 ▶); Szabó *et al.* (2008 ▶); Xie *et al.* (2008 ▶). For a related structure, see: Zhang *et al.* (2008 ▶).
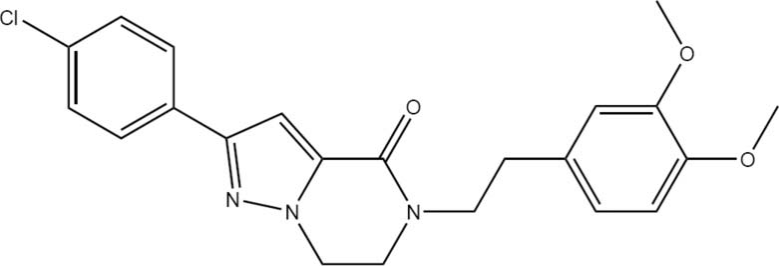


## Experimental

### 

#### Crystal data

C_22_H_22_ClN_3_O_3_
*M* *_r_* = 411.88
Triclinic, 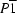

*a* = 7.1709 (4) Å
*b* = 10.6982 (5) Å
*c* = 13.9169 (6) Å
α = 81.156 (3)°
β = 77.150 (2)°
γ = 72.278 (2)°
*V* = 987.25 (8) Å^3^
*Z* = 2
Mo *K*α radiation
μ = 0.22 mm^−1^
*T* = 293 K
0.40 × 0.20 × 0.10 mm

#### Data collection

Bruker SMART CCD area-detector diffractometer
Absorption correction: multi-scan (*SADABS*; Bruker 2005 ▶) *T*_min_ = 0.916, *T*_max_ = 0.978
8654 measured reflections
3999 independent reflections
2969 reflections with *I* > 2σ(*I*)
*R*_int_ = 0.021

#### Refinement

*R*[*F* ^2^ > 2σ(*F* ^2^)] = 0.041
*wR*(*F* ^2^) = 0.115
*S* = 1.03
3999 reflections
326 parameters
H atoms treated by a mixture of independent and constrained refinement
Δρ_max_ = 0.20 e Å^−3^
Δρ_min_ = −0.30 e Å^−3^

### 

Data collection: *SMART* (Bruker, 2005 ▶); cell refinement: *SAINT* (Bruker, 2005 ▶); data reduction: *SAINT*; program(s) used to solve structure: *SHELXS97* (Sheldrick, 2008 ▶); program(s) used to refine structure: *SHELXL97* (Sheldrick, 2008 ▶); molecular graphics: *XP* in *SHELXTL* (Sheldrick, 2008 ▶); software used to prepare material for publication: *SHELXL97*.

## Supplementary Material

Crystal structure: contains datablocks I, global. DOI: 10.1107/S1600536809020212/pv2160sup1.cif

Structure factors: contains datablocks I. DOI: 10.1107/S1600536809020212/pv2160Isup2.hkl

Additional supplementary materials:  crystallographic information; 3D view; checkCIF report

## Figures and Tables

**Table 1:** Hydrogen-bond geometry (Å, °)

*D*—H⋯*A*	*D*—H	H⋯*A*	*D*⋯*A*	*D*—H⋯*A*
C5—H5⋯O1^i^	0.92 (2)	2.48 (2)	3.341 (2)	156.3 (17)
C8—H8⋯O1^i^	0.94 (2)	2.39 (2)	3.296 (2)	161.4 (19)
C11—H11*B*⋯*Cg*1^ii^	0.99 (2)	2.67 (2)	3.413 (2)	132.2 (16)
C12—H12*A*⋯*Cg*2^iii^	1.00 (3)	2.77 (2)	3.640 (2)	146.2 (17)

Symmetry codes: (i) 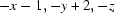; (ii) 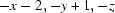; (iii) 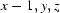. *Cg*1 and *Cg*2 are centroids of the C15–C20 and C1–C6 rings, respectively.

**Fig. 1.**
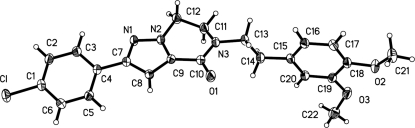
The molecular structure of the title compound showing displacement ellipsoids drawn at 30% probability level.

## supplementary crystallographic information

### Comment

Many pyrazole derivatives are known to exhibit a wide range of biological properties (Farag *et al.*, 2008; Szabó *et al.*, 2008). As part of our continuing project on the study of the interactions occurring between small molecules and proteins (Pan *et al.*, 2008; Xie *et al.*, 2008; Zhang *et al.*, 2008), we report here the crystal structure of the title compound.

The molecular structure of the title compound is illustrated in Fig. 1. In contrast to our previously reported structure of a related compound (Zhang *et al.*, 2008), the conformation of the six-membered heterocyclic ring (N2/N3/C9—C12) in the title compound is close to a screw-boat, with atoms C11 and N3 out of the plane of the remaining four atoms by 0.681 (2) and 0.214 (2) Å, respectively. In the crystal structure, the dihedral angles of the phenyl rings (C1—C6) and (C15—C20) with the pyrazol ring (N1/N2/C7—C9) are 16.05 (10) and 84.84 (10)°, respectively. The crystal packing is stabilized by intermolecular C—H···O interactions and is further consolidated by C—H···π interactions (Table 1).

### Experimental

A solution containing ethyl-3-(4-chlorophenyl)-1-(2-bromoethyl)-1*H*-pyrazole-5-carboxylate (146 mg, 0.4 mmol), 2-(3,4-dimethoxyphenyl)ethanamine (724 mg, 4.0 mmol) and potassium iodide (13 mg, 0.08 mmol) in acetonitrile (10 ml) was refluxed under nitrogen for 3 h. Then the mixture was cooled, filtered, and the solvent was removed under reduced pressure. The product was obtained in 57% yield by column chromatography on silica gel using ethyl acetate as eluent. Crystals suitable for X-ray diffraction were obtained by slow evaporation of a solution of the solid dissolved in ethyl acetate at room temperature for 5 days.

### Refinement

The H atoms were located in difference Fourier maps, their positional and isotropic vibrational parameters were refined freely except for the methyl H-atoms which were included in the refinements at geometrically idealized positions in riding mode with C–H = 0.96 Å and U_iso_(H) = 1.5 U_eq_(C).

### Crystal data

**Table:**

C_22_H_22_ClN_3_O_3_	*Z* = 2
*M**_r_* = 411.88	*F*(000) = 432
Triclinic, *P*1	*D*_x_ = 1.386 Mg m^−^^3^
Hall symbol: -P 1	Mo *K*α radiation, λ = 0.71073 Å
*a* = 7.1709 (4) Å	Cell parameters from 2906 reflections
*b* = 10.6982 (5) Å	θ = 3.0–26.1°
*c* = 13.9169 (6) Å	µ = 0.22 mm^−^^1^
α = 81.156 (3)°	*T* = 293 K
β = 77.150 (2)°	Block, colorless
γ = 72.278 (2)°	0.40 × 0.20 × 0.10 mm
*V* = 987.25 (8) Å^3^	

### Data collection

**Table:**

Bruker SMART CCD area-detector diffractometer	3999 independent reflections
Radiation source: fine-focus sealed tube	2969 reflections with *I* > 2σ(*I*)
graphite	*R*_int_ = 0.021
φ and ω scans	θ_max_ = 26.4°, θ_min_ = 2.0°
Absorption correction: multi-scan (*SADABS*; Bruker 2005)	*h* = −7→8
*T*_min_ = 0.916, *T*_max_ = 0.978	*k* = −13→13
8654 measured reflections	*l* = −17→17

### Refinement

**Table:**

Refinement on *F*^2^	Primary atom site location: structure-invariant direct methods
Least-squares matrix: full	Secondary atom site location: difference Fourier map
*R*[*F*^2^ > 2σ(*F*^2^)] = 0.041	Hydrogen site location: inferred from neighbouring sites
*wR*(*F*^2^) = 0.115	H atoms treated by a mixture of independent and constrained refinement
*S* = 1.03	*w* = 1/[σ^2^(*F*_o_^2^) + (0.0564*P*)^2^ + 0.1941*P*] where *P* = (*F*_o_^2^ + 2*F*_c_^2^)/3
3999 reflections	(Δ/σ)_max_ < 0.001
326 parameters	Δρ_max_ = 0.20 e Å^−^^3^
0 restraints	Δρ_min_ = −0.30 e Å^−^^3^

### Special details

**Table:**

Geometry. All e.s.d.'s (except the e.s.d. in the dihedral angle between two l.s. planes) are estimated using the full covariance matrix. The cell e.s.d.'s are taken into account individually in the estimation of e.s.d.'s in distances, angles and torsion angles; correlations between e.s.d.'s in cell parameters are only used when they are defined by crystal symmetry. An approximate (isotropic) treatment of cell e.s.d.'s is used for estimating e.s.d.'s involving l.s. planes.
Refinement. Refinement of *F*^2^ against ALL reflections. The weighted *R*-factor *wR* and goodness of fit *S* are based on *F*^2^, conventional *R*-factors *R* are based on *F*, with *F* set to zero for negative *F*^2^. The threshold expression of *F*^2^ > σ(*F*^2^) is used only for calculating *R*-factors(gt) *etc*. and is not relevant to the choice of reflections for refinement. *R*-factors based on *F*^2^ are statistically about twice as large as those based on *F*, and *R*- factors based on ALL data will be even larger.

### Fractional atomic coordinates and isotropic or equivalent isotropic displacement parameters (Å^2^)

**Table:**

	*x*	*y*	*z*	*U*_iso_*/*U*_eq_	
Cl	0.20421 (8)	0.95549 (6)	0.40014 (4)	0.06084 (19)	
N1	−0.4397 (2)	0.70036 (14)	0.26041 (11)	0.0390 (4)	
N2	−0.5553 (2)	0.69103 (14)	0.19947 (10)	0.0367 (4)	
N3	−0.7921 (2)	0.70588 (15)	0.06451 (11)	0.0390 (4)	
O1	−0.7274 (2)	0.89705 (14)	−0.00841 (10)	0.0575 (4)	
O2	−1.3542 (2)	0.72159 (14)	−0.37111 (10)	0.0533 (4)	
O3	−0.9862 (2)	0.58773 (15)	−0.41075 (10)	0.0544 (4)	
C1	0.0314 (3)	0.91488 (18)	0.34943 (14)	0.0406 (4)	
C2	−0.0785 (3)	0.8341 (2)	0.40461 (15)	0.0450 (5)	
H2	−0.065 (3)	0.8025 (19)	0.4699 (15)	0.048 (6)*	
C3	−0.2109 (3)	0.79940 (19)	0.36251 (14)	0.0419 (4)	
H3	−0.289 (3)	0.744 (2)	0.3994 (15)	0.057 (6)*	
C4	−0.2335 (3)	0.84383 (17)	0.26496 (12)	0.0343 (4)	
C5	−0.1236 (3)	0.92790 (18)	0.21187 (14)	0.0390 (4)	
H5	−0.135 (3)	0.9556 (19)	0.1468 (15)	0.046 (5)*	
C6	0.0084 (3)	0.96339 (19)	0.25367 (14)	0.0411 (4)	
H6	0.082 (3)	1.019 (2)	0.2150 (15)	0.049 (6)*	
C7	−0.3707 (3)	0.80384 (17)	0.21908 (12)	0.0345 (4)	
C8	−0.4449 (3)	0.86047 (19)	0.13250 (13)	0.0367 (4)	
H8	−0.421 (3)	0.933 (2)	0.0897 (15)	0.047 (6)*	
C9	−0.5643 (3)	0.78601 (17)	0.12271 (12)	0.0344 (4)	
C10	−0.6987 (3)	0.80106 (19)	0.05288 (13)	0.0386 (4)	
C11	−0.7288 (4)	0.5823 (2)	0.12678 (16)	0.0499 (5)	
H11B	−0.842 (3)	0.543 (2)	0.1422 (16)	0.058 (6)*	
H11A	−0.600 (4)	0.519 (2)	0.0875 (17)	0.066 (7)*	
C12	−0.6833 (4)	0.6042 (2)	0.22192 (15)	0.0462 (5)	
H12B	−0.611 (3)	0.522 (2)	0.2536 (15)	0.053 (6)*	
H12A	−0.811 (4)	0.647 (2)	0.2650 (17)	0.069 (7)*	
C13	−0.9414 (3)	0.7191 (2)	0.00409 (15)	0.0424 (5)	
H13B	−1.051 (3)	0.684 (2)	0.0467 (15)	0.052 (6)*	
C14	−0.8546 (3)	0.6485 (3)	−0.08922 (16)	0.0488 (5)	
H14B	−0.807 (4)	0.551 (3)	−0.0693 (18)	0.073 (7)*	
H14A	−0.736 (4)	0.676 (2)	−0.1225 (17)	0.066 (7)*	
C15	−0.9982 (3)	0.67175 (18)	−0.16026 (13)	0.0383 (4)	
C16	−1.1963 (3)	0.7426 (2)	−0.13969 (14)	0.0416 (4)	
H16	−1.255 (3)	0.777 (2)	−0.0773 (16)	0.056 (6)*	
C17	−1.3206 (3)	0.7617 (2)	−0.20844 (15)	0.0429 (5)	
H17	−1.458 (3)	0.814 (2)	−0.1909 (14)	0.047 (6)*	
C18	−1.2466 (3)	0.70993 (17)	−0.29847 (14)	0.0392 (4)	
C19	−1.0451 (3)	0.63712 (18)	−0.32041 (13)	0.0387 (4)	
C20	−0.9246 (3)	0.61940 (19)	−0.25182 (14)	0.0399 (4)	
H20	−0.793 (4)	0.570 (2)	−0.2684 (15)	0.056 (6)*	
C21	−1.5441 (3)	0.8157 (2)	−0.36227 (18)	0.0613 (6)	
H21A	−1.6042	0.8145	−0.4172	0.092*	
H21B	−1.5293	0.9018	−0.3621	0.092*	
H21C	−1.6275	0.7944	−0.3016	0.092*	
C22	−0.7782 (3)	0.5470 (2)	−0.45031 (16)	0.0593 (6)	
H22A	−0.7578	0.5147	−0.5136	0.089*	
H22B	−0.7124	0.4782	−0.4060	0.089*	
H22C	−0.7243	0.6205	−0.4579	0.089*	
H13A	−0.998 (3)	0.813 (2)	−0.0122 (14)	0.048 (6)*	

### Atomic displacement parameters (Å^2^)

**Table:**

	*U*^11^	*U*^22^	*U*^33^	*U*^12^	*U*^13^	*U*^23^
Cl	0.0547 (4)	0.0822 (4)	0.0621 (4)	−0.0307 (3)	−0.0219 (3)	−0.0163 (3)
N1	0.0449 (9)	0.0398 (8)	0.0395 (8)	−0.0162 (7)	−0.0192 (7)	0.0006 (7)
N2	0.0410 (9)	0.0385 (8)	0.0365 (8)	−0.0167 (7)	−0.0151 (7)	0.0016 (6)
N3	0.0417 (9)	0.0462 (9)	0.0368 (8)	−0.0195 (7)	−0.0158 (7)	−0.0003 (7)
O1	0.0699 (10)	0.0651 (9)	0.0521 (8)	−0.0378 (8)	−0.0347 (8)	0.0235 (7)
O2	0.0527 (9)	0.0580 (9)	0.0527 (8)	−0.0050 (7)	−0.0288 (7)	−0.0097 (7)
O3	0.0521 (9)	0.0692 (10)	0.0418 (8)	−0.0064 (8)	−0.0151 (7)	−0.0174 (7)
C1	0.0382 (11)	0.0423 (10)	0.0462 (11)	−0.0098 (9)	−0.0149 (8)	−0.0116 (8)
C2	0.0546 (13)	0.0505 (11)	0.0368 (10)	−0.0187 (10)	−0.0192 (9)	−0.0009 (9)
C3	0.0489 (12)	0.0426 (11)	0.0398 (10)	−0.0193 (10)	−0.0133 (9)	0.0003 (8)
C4	0.0354 (10)	0.0353 (9)	0.0345 (9)	−0.0091 (8)	−0.0114 (8)	−0.0043 (7)
C5	0.0416 (11)	0.0415 (10)	0.0356 (10)	−0.0121 (9)	−0.0123 (8)	−0.0002 (8)
C6	0.0412 (11)	0.0422 (11)	0.0442 (11)	−0.0172 (9)	−0.0086 (9)	−0.0045 (8)
C7	0.0352 (10)	0.0355 (9)	0.0344 (9)	−0.0103 (8)	−0.0088 (8)	−0.0040 (7)
C8	0.0384 (10)	0.0402 (10)	0.0357 (9)	−0.0168 (9)	−0.0116 (8)	0.0021 (8)
C9	0.0353 (10)	0.0379 (9)	0.0319 (9)	−0.0126 (8)	−0.0093 (7)	0.0001 (7)
C10	0.0391 (11)	0.0468 (11)	0.0344 (10)	−0.0174 (9)	−0.0104 (8)	−0.0007 (8)
C11	0.0605 (15)	0.0477 (12)	0.0535 (12)	−0.0270 (12)	−0.0243 (11)	0.0039 (10)
C12	0.0566 (14)	0.0456 (12)	0.0471 (12)	−0.0281 (11)	−0.0209 (11)	0.0083 (10)
C13	0.0391 (11)	0.0545 (13)	0.0415 (11)	−0.0187 (10)	−0.0145 (9)	−0.0061 (9)
C14	0.0400 (12)	0.0676 (15)	0.0436 (11)	−0.0146 (11)	−0.0127 (9)	−0.0133 (10)
C15	0.0396 (11)	0.0434 (10)	0.0385 (10)	−0.0179 (9)	−0.0109 (8)	−0.0055 (8)
C16	0.0408 (11)	0.0498 (11)	0.0392 (10)	−0.0164 (9)	−0.0083 (9)	−0.0109 (9)
C17	0.0360 (11)	0.0456 (11)	0.0495 (11)	−0.0111 (9)	−0.0101 (9)	−0.0089 (9)
C18	0.0442 (11)	0.0368 (10)	0.0434 (10)	−0.0155 (9)	−0.0189 (9)	0.0003 (8)
C19	0.0448 (11)	0.0401 (10)	0.0346 (9)	−0.0140 (9)	−0.0116 (8)	−0.0029 (8)
C20	0.0374 (11)	0.0433 (11)	0.0407 (10)	−0.0108 (9)	−0.0102 (9)	−0.0060 (8)
C21	0.0494 (13)	0.0657 (15)	0.0729 (15)	−0.0083 (12)	−0.0315 (12)	−0.0051 (12)
C22	0.0617 (16)	0.0631 (14)	0.0478 (12)	−0.0061 (12)	−0.0090 (11)	−0.0142 (10)

### Geometric parameters (Å, °)

**Table:**

Cl—C1	1.7416 (18)	C9—C10	1.474 (2)
N1—N2	1.3439 (19)	C11—C12	1.499 (3)
N1—C7	1.346 (2)	C11—H11B	0.99 (2)
N2—C9	1.355 (2)	C11—H11A	1.06 (2)
N2—C12	1.452 (2)	C12—H12B	0.97 (2)
N3—C10	1.352 (2)	C12—H12A	1.00 (3)
N3—C13	1.465 (2)	C13—C14	1.516 (3)
N3—C11	1.470 (2)	C13—H13B	1.01 (2)
O1—C10	1.227 (2)	C13—H13A	0.97 (2)
O2—C18	1.372 (2)	C14—C15	1.523 (2)
O2—C21	1.418 (3)	C14—H14B	1.01 (3)
O3—C19	1.368 (2)	C14—H14A	0.98 (2)
O3—C22	1.425 (3)	C15—C16	1.380 (3)
C1—C2	1.378 (3)	C15—C20	1.399 (3)
C1—C6	1.380 (3)	C16—C17	1.401 (3)
C2—C3	1.383 (3)	C16—H16	0.95 (2)
C2—H2	0.94 (2)	C17—C18	1.377 (3)
C3—C4	1.396 (2)	C17—H17	0.97 (2)
C3—H3	0.95 (2)	C18—C19	1.407 (3)
C4—C5	1.394 (3)	C19—C20	1.380 (2)
C4—C7	1.476 (2)	C20—H20	0.93 (2)
C5—C6	1.382 (3)	C21—H21A	0.9600
C5—H5	0.92 (2)	C21—H21B	0.9600
C6—H6	0.95 (2)	C21—H21C	0.9600
C7—C8	1.402 (2)	C22—H22A	0.9600
C8—C9	1.374 (2)	C22—H22B	0.9600
C8—H8	0.94 (2)	C22—H22C	0.9600
			
N2—N1—C7	104.41 (13)	C11—C12—H12B	110.9 (12)
N1—N2—C9	112.51 (14)	N2—C12—H12A	109.6 (14)
N1—N2—C12	123.74 (14)	C11—C12—H12A	108.3 (13)
C9—N2—C12	122.94 (15)	H12B—C12—H12A	112.2 (19)
C10—N3—C13	119.09 (15)	N3—C13—C14	112.60 (17)
C10—N3—C11	121.19 (15)	N3—C13—H13B	107.6 (12)
C13—N3—C11	119.25 (15)	C14—C13—H13B	111.3 (12)
C18—O2—C21	117.38 (16)	N3—C13—H13A	107.0 (12)
C19—O3—C22	118.05 (15)	C14—C13—H13A	110.7 (12)
C2—C1—C6	120.98 (17)	H13B—C13—H13A	107.4 (17)
C2—C1—Cl	119.85 (14)	C13—C14—C15	114.67 (18)
C6—C1—Cl	119.16 (15)	C13—C14—H14B	108.3 (14)
C1—C2—C3	119.31 (18)	C15—C14—H14B	109.1 (14)
C1—C2—H2	121.1 (13)	C13—C14—H14A	108.0 (14)
C3—C2—H2	119.6 (13)	C15—C14—H14A	110.3 (13)
C2—C3—C4	121.06 (19)	H14B—C14—H14A	106 (2)
C2—C3—H3	120.8 (13)	C16—C15—C20	118.18 (17)
C4—C3—H3	118.2 (13)	C16—C15—C14	123.66 (17)
C5—C4—C3	118.22 (16)	C20—C15—C14	118.15 (17)
C5—C4—C7	120.68 (15)	C15—C16—C17	121.03 (18)
C3—C4—C7	121.10 (16)	C15—C16—H16	121.5 (13)
C6—C5—C4	120.96 (17)	C17—C16—H16	117.4 (13)
C6—C5—H5	120.4 (12)	C18—C17—C16	120.32 (19)
C4—C5—H5	118.6 (12)	C18—C17—H17	122.1 (12)
C1—C6—C5	119.42 (18)	C16—C17—H17	117.5 (12)
C1—C6—H6	121.9 (12)	O2—C18—C17	125.43 (18)
C5—C6—H6	118.7 (12)	O2—C18—C19	115.24 (16)
N1—C7—C8	111.40 (15)	C17—C18—C19	119.32 (17)
N1—C7—C4	120.36 (15)	O3—C19—C20	125.34 (18)
C8—C7—C4	128.23 (16)	O3—C19—C18	115.07 (15)
C9—C8—C7	104.71 (16)	C20—C19—C18	119.59 (17)
C9—C8—H8	126.3 (12)	C19—C20—C15	121.57 (19)
C7—C8—H8	129.0 (12)	C19—C20—H20	117.2 (13)
N2—C9—C8	106.96 (15)	C15—C20—H20	121.2 (13)
N2—C9—C10	120.98 (15)	O2—C21—H21A	109.5
C8—C9—C10	131.84 (16)	O2—C21—H21B	109.5
O1—C10—N3	123.38 (16)	H21A—C21—H21B	109.5
O1—C10—C9	121.11 (16)	O2—C21—H21C	109.5
N3—C10—C9	115.44 (15)	H21A—C21—H21C	109.5
N3—C11—C12	112.58 (17)	H21B—C21—H21C	109.5
N3—C11—H11B	106.1 (12)	O3—C22—H22A	109.5
C12—C11—H11B	108.8 (12)	O3—C22—H22B	109.5
N3—C11—H11A	109.6 (12)	H22A—C22—H22B	109.5
C12—C11—H11A	108.6 (12)	O3—C22—H22C	109.5
H11B—C11—H11A	111.2 (18)	H22A—C22—H22C	109.5
N2—C12—C11	108.36 (16)	H22B—C22—H22C	109.5
N2—C12—H12B	107.4 (13)		

### Hydrogen-bond geometry (Å, °)

**Table:**

*D*—H···*A*	*D*—H	H···*A*	*D*···*A*	*D*—H···*A*
C5—H5···O1^i^	0.92 (2)	2.48 (2)	3.341 (2)	156.3 (17)
C8—H8···O1^i^	0.94 (2)	2.39 (2)	3.296 (2)	161.4 (19)
C11—H11B···Cg1^ii^	0.99 (2)	2.67 (2)	3.413 (2)	132.2 (16)
C12—H12A···Cg2^iii^	1.00 (3)	2.77 (2)	3.640 (2)	146.2 (17)
C21—H21B···Cg2^iv^	0.96	2.98	3.531 (2)	117

Symmetry codes: (i) −*x*−1, −*y*+2, −*z*; (ii) −*x*−2, −*y*+1, −*z*; (iii) *x*−1, *y*, *z*; (iv) −*x*−2, −*y*+2, −*z*.
